# Field metabolic rates of teleost fishes are recorded in otolith carbonate

**DOI:** 10.1038/s42003-018-0266-5

**Published:** 2019-01-18

**Authors:** Ming-Tsung Chung, Clive N. Trueman, Jane Aanestad Godiksen, Mathias Engell Holmstrup, Peter Grønkjær

**Affiliations:** 1 Department of Bioscience, Section for Aquatic Biology, Aarhus University, 8000 Aarhus C, Denmark; 2 Ocean and Earth Science, University of Southampton Waterfront Campus, European Way, Southampton, SO14 3ZH UK; 3 Institute of Marine Research, Postbox 1870 Nordnes, 5817 Bergen, Norway

## Abstract

Field metabolic rate (FMR) is key to understanding individual and population-level responses to environmental changes, but is challenging to measure in field conditions, particularly in aquatic environments. Here we show that FMR can be estimated directly from the isotopic composition of carbon in fish otoliths (δ^13^C_oto_). We describe the relationship between δ^13^C_oto_ values and oxygen consumption rate, and report results from laboratory experiments relating individual-level measurements of oxygen consumption rates to δ^13^C_oto_ values in Atlantic cod (*Gadus morhua*). We apply our new δ^13^C_oto_ metabolic proxy to existing δ^13^C_oto_ data from wild cod and four deepwater fish species to test the validity of inferred FMR estimates. The δ^13^C_oto_ metabolic proxy offers a new approach to study physiological ecology in free-ranging wild fishes. Otolith-based proxies for FMR are particularly promising as they allow retrospective assessment of time-integrated, individual-level FMR throughout an individual fish’s life history.

## Abstract (web summary)

Ming-Tsung Chung et al. report a method for estimating field metabolic rate (FMR) in teleost fishes using the isotopic composition of carbon found in inner ear structures called otoliths. They show that their method can provide accurate estimates of FMR for free-ranging wild fishes and allows for tracking FMR of individual fish over time.

## Introduction

Predicting ecosystem, species and population responses to environmental change is one of the most pressing ecological challenges of our time. Resilience or adaptation to changing conditions can be provided by plastic behavioural and physiological responses of individuals, such as thermal acclimation and habitat shifts^1,2^. In aquatic ecosystems, the identification of adaptation and physiological responses is hampered by the challenges related to monitoring the relevant physiological variables in free living organisms^3,4^.

Several classic and emerging topics in fish ecology and conservation physiology highlight the need for improved measurements of key physiological variables in free-ranging fishes. The metabolic rate of poikilotherms is strongly dependent on temperature and body mass^5,6^, but at the same time there is large variation among individuals that experience the same environmental conditions. This variation has been linked to heritable and genetic differences among individuals^7^, and is expressed in the life history and in “consistent individual differences (CID’s)” or personalities, such as aggressiveness and predator avoidance^8^. The relationship between metabolic rate and fitness components changes shape in response to environmental conditions, such as food level. For example, with abundant food supplies, active individuals with higher metabolic rates have been shown to exhibit higher growth rates than less active individuals^9^. On the other hand, low metabolic rates may favour animals during periods with lower food availability^10,11^. A population may therefore have more resilience to environmental change if the individuals exhibit a diversity of metabolic phenotypes in the wild^2^.

The resilience of fish populations to environmental change, especially climate and fisheries, is dependent on the growth and reproductive potential of the populations. The amount of energy directed towards these fitness components is the difference between assimilated energy and energy spent on metabolism, the field metabolic rate (FMR)^8^. Hence, environmental changes that affect FMR are likely to have a direct impact on the productivity of fish populations. Finally, global change is expected to lead to major redistribution and reorganisation of marine fish communities. Increasing ocean temperatures are thought to shift communities towards higher latitudes to allow them to remain within their preferred environmental niches^12^. Reliable measurements of FMR are needed for accurate predictions of these phenomena.

**Fig. 1:**
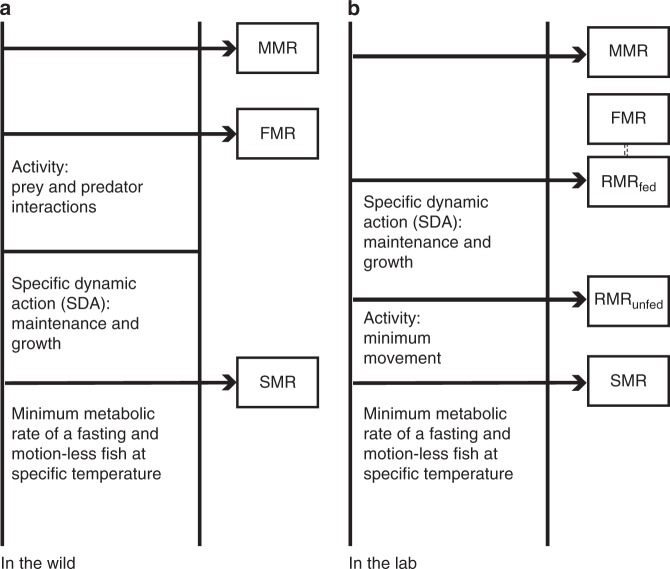
Definition of terms used to describe aspects of fish metabolism and their inter-relationships under **a** wild and **b** lab-controlled environments. SMR is standard metabolic rate, which is calculated as the effective metabolic rate at swimming speed 0 extrapolated from a series of measurements of oxygen consumption at different swimming speeds. Routine metabolic rate (RMR) is an average of daily oxygen consumption, which may include feeding or no feeding between studies. FMR and MMR are field metabolic rate and maximum metabolic rate, respectively

FMR is the sum of three metabolic components: standard metabolic rate (SMR), specific dynamic action (SDA, the postprandial increase in metabolism) and activity metabolism (Fig. 1a). The aerobic scope, the difference between the maximal metabolic rate and the standard metabolic rate (MMR-SMR) or the factorial equivalent (MMR/SMR) are expressions of the potential aerobic performance of an individual under a given set of environmental conditions. In contrast, the FMR-SMR or FMR/SMR expresses how much of this aerobic scope is actually used by wild fish in their natural habitats and is important in order to evaluate potential energy constraints under different environmental conditions and climate scenarios^13^. Finally, estimates of FMR serve to constrain the very uncertain estimates of SDA and activity metabolism in the field (Fig. 1a).

**Fig. 2:**
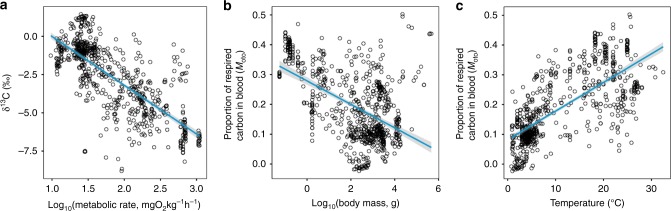
Among species variations in δ^13^C_oto_ values and metabolism. **a** Published δ^13^C_oto_ values in 76 species covary negatively with estimates of standard metabolic rate predicted by metabolic theory. **b** The proportion of respired carbon in otolith aragonite (*M*_oto_) estimated from δ^13^C_oto_ values decreases with logarithmic body mass. **c** *M*_oto_ values increase with environmental temperature. (linear regression, temperature: *t* value = 27.0, *p* < 0.001, *R*^2^ = 0.512; logarithmic body mass: *t* value = −16.0, *p* < 0.001, *R*^2^ = 0.267)

If we want to understand relationships between fitness, behaviour and a complex environment, time-integrated individual-level FMR is the ecologically and evolutionarily relevant trait to study^8,14,15^. Unfortunately, FMR is challenging to measure. In terrestrial animals, the doubly labelled water technique has been widely used to estimate CO_2_ production rates by detecting the difference in the isotope elimination rates of deuterium and ^18^O in CO_2_ and H_2_O^3,16^. However, this method cannot be applied to fish due to the higher body water turnover rates resulting in an inaccurate estimation of CO_2_ production^15^. Several indirect approaches to estimate FMR in fishes^15^ have been suggested and the isotopic composition of carbon in otoliths appears particularly promising (Fig. 2)^17–20^. However, to date there has been no systematic attempt to link otolith tracer-based measurements of FMR directly to other estimates of metabolic rate such as oxygen consumption rates.

The stable isotope composition of carbon in the aragonitic mineral component of fish otoliths (here termed δ^13^C_oto_ value) varies with the isotopic composition of dissolved carbon in blood and has been proposed as a biogeochemical proxy of mass-specific field metabolism^17–20^. Dissolved carbon in fish blood is derived from two sources: dissolved inorganic carbon (DIC) from the ambient water and metabolic carbon released from the respiration of food^17,19,21^. These two carbon sources are isotopically distinct, as δ^13^C values in DIC are typically more than 15 per mille higher than δ^13^C values of metabolic carbon^17,22^. The isotopic composition of dissolved carbon in fish blood, and consequently in the otolith carbonate, is therefore a weighted average of the δ^13^C values of DIC and metabolic carbon. Total carbonate concentrations in blood are physiologically controlled to regulate body fluid pH, so as respiration rates increase, the proportion of metabolic (respiratory) carbon in blood increases, and blood δ^13^C values decrease^17^. The isotopic composition of carbon in fish otoliths is therefore linked to oxygen consumption though metabolic oxidation of dietary carbon.

An advantage of using otoliths as field metabolic recorders is that they are inert acellular structures with no metabolic reworking or turnover. The otolith therefore contains an uninterrupted structural record of body size and growth rate in the form of daily and annual growth increments, as well as a chemical record of ambient water temperature, fish diet and internal (physiological) conditions^23^.

However, while the otolith carbon isotope proxy for FMR has theoretical and empirical support, there is currently a lack of experimental validation quantifying the scaling between δ^13^C_oto_ values and oxygen consumption rates, significantly limiting the utility of δ^13^C_oto_ values in wider metabolic ecology. Here we demonstrate that δ^13^C_oto_ values are effective proxies for the rate of oxygen consumption fuelling FMR in marine fishes using two approaches. First, we document the potential of the otolith isotope proxy through a laboratory-controlled rearing and calibration experiment with Atlantic cod (*Gadus morhua*), and establish the relationship between the carbon isotope proxy and oxygen consumption rate. We then strengthen the confidence in the δ^13^C FMR proxy by demonstrating that it complies with estimates of the metabolic power budget and the metabolic-level boundaries hypothesis (MLBH)^24–27^.

## Results

### Oxygen consumption and *M*_oto_ of reared Atlantic cod

**Fig. 3:**
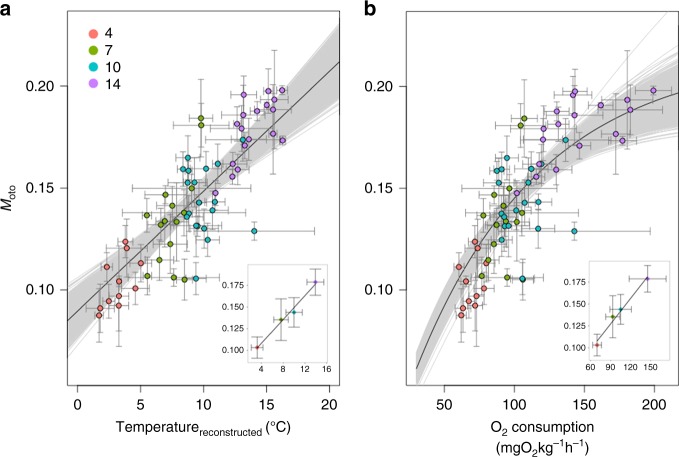
The relationship between the proportion of metabolic carbon in otolith aragonite (*M*_oto_) and **a** temperature and **b** oxygen consumption. Temperature experienced by individual fish is reconstructed from measured otolith δ^18^O values. The O_2_ consumption is estimated as routine metabolic rate with feeding conditions. The grey lines reflect 10,000 Monte Carlo simulation models considering uncertainty in both *x* and *y* variables

In our controlled rearing experiment, we employed four temperature groups (4, 7, 10 and 14 °C). Average body masses were similar between groups and temperature was the only variable exerting a significant influence on the proportion of metabolically derived carbon in otolith carbonate (*M*_oto_) (two-way ANOVA, *n* = 80; temperature: *t* value = 6.12, *p* < 0.01; body mass: *t* value = 0.889, *p* = 0.378, temperature-body mass: *t* value = −1.07, *p* = 0.289). Linear regression modelling demonstrated that temperature alone explained 72.6% of the variation in the proportion of metabolically derived carbon in otolith carbonate (*M*_oto_) (*n* = 63, *t* value = 12.9, *p* < 0.001) which increased linearly by approximately 0.007 per 1 °C of temperature increase (Fig. 3a).

Predictions of individual-level SMR drawn the from metabolic theory of ecology (MTE) based on body mass and temperature^5,28^ (see Supplementary Note 1 for details) co-varied closely with measured oxygen consumption rates in 17 reared Atlantic cod individuals (linear regression model: *y* = 1.43 × 10^13^*x*, *t* = 8.59, *p* < 0.01, *R*^2^ = 0.82). Accordingly, we used individual body mass and temperature to predict oxygen consumption rates (SMR) for a further 63 individuals from the rearing experiment for which no direct measurements of oxygen consumption rates were available. In a laboratory-controlled environment with ad libitum feeding, cod individuals are assumed to have little energetic demand associated with predator avoidance and feeding. FMR of cod in the rearing tank is therefore assumed to be close to RMR_fed_ (routine metabolic rate, in which the energy used for maintenance, regular activity and food digestion) (Fig. 1b). Based on data in Jordan et al.^29^ the RMR_fed_ was estimated to be 1.55(±0.0832) × SMR. We modelled the relationship between the proportion of metabolically derived carbon in otolith carbonate and RMR_fed_ expressed as oxygen consumption rate as an increasing exponential decay model, because the proportional *M*_oto_ term is constrained by both upper and lower boundaries.

1 $$M_{{\mathrm {oto}}} = C\left( {1 - e^{ - k\left( {{\mathrm {oxygen}}\,{\mathrm {consumption}}} \right)}} \right),$$

where *C* is the upper bound and *k* is the decay constant. The best fit relationship between the proportion of metabolic carbon in otolith aragonite and modelled oxygen consumption rate gave a decay constant and an upper bound of 8.80×10^−3^ ± 1.38×10^−3^ (*t* = 6.36, *p* < 0.001) and 0.243 ± 0.035 (*t* = 10.9, *p* < 0.001), respectively (Fig. 3b). The maximum contribution of metabolic-derived carbon predicted for the Atlantic cod was around 24%, which is close to, but slightly lower than published estimates^17^.

We constrained the precision associated with estimating FMR from *M*_oto_ values by evaluating repeated measurements of the same otolith. This revealed 0.17‰ intra-individual variation (standard deviation) of δ^13^C_oto_ values_,_ probably reflecting analytical error and differing effective temporal resolution between samples taken from different areas of the otolith. In the laboratory experiments, the tank water δ^13^C values and diet δ^13^C values (diet δ^13^C values were estimated from the otolith organic material) yielded standard deviation of 0.035−0.019‰ and 0.86‰, respectively. Including these variations, the uncertainty (standard deviation) of the *M*_oto_ term was on average 0.010 (*n* = 63) and less than 0.025. Variation in the observed *M*_oto_ term among individual fish in similar temperature conditions and of comparable body size was larger than the estimated analytical uncertainty. For example, among individuals *M*_oto_ values ranged from 0.11 to 0.18 at 7 °C and 10 °C respectively, but the variation was smaller in the 14 °C (0.15–0.20) and 4 °C (0.09–0.12) groups. In laboratory conditions, among-individual variation in *M*_oto_ terms therefore revealed individual-level variability in metabolic performance and may indicate the scope for recovering inter-individual metabolic differences within a given environmental setting. Based on the assessment of uncertainty, to recover metabolic differences between two hypothetical cod populations experiencing a 2 °C difference in temperature (equal to ca. 17% difference in estimated oxygen consumption rates) with 80% test power would require a minimum of 32 samples per population (Supplementary Note 1).

We strengthened the confidence in the δ^13^C_oto_ FMR proxy by comparing otolith-derived estimates of FMR to literature-derived ranges of the metabolic components of fish. We devised five tests for our FMR proxy.

### FMR compared to the metabolic power budget (Tests 1−3)

**Fig. 4:**
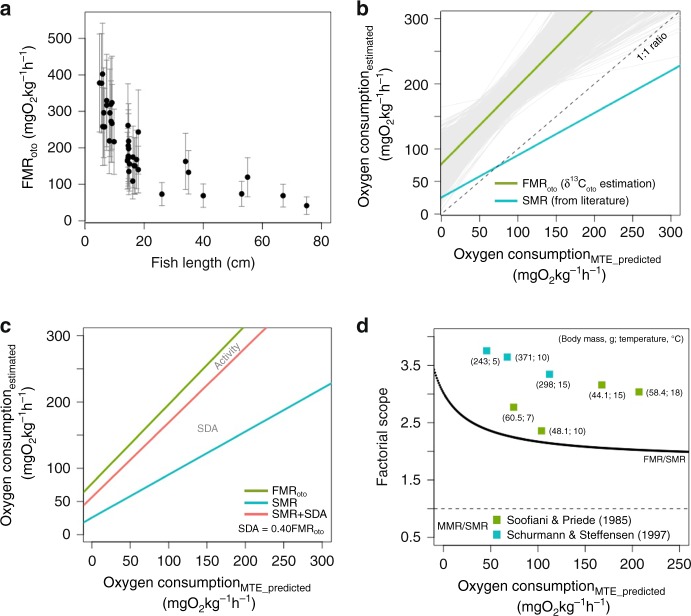
**a** FMR_oto_ estimated from wild cod individuals based on the relationship built from reared cod in this study. **b** A comparison between FMR_oto_ and literature values of SMR (for individual values see Supplementary Note 1) against the SMR predicted by the MTE. The grey area represents 10,000 times of Monte Carlo simulation in FMR_oto_ and the green line is the average (Test 1). **c** Proportion of three components in FMR_oto_ that is determined by an SDA/FMR_oto_ ratio value of 0.4. The ratio is a maximum value of SDA proportional to FMR (Test 2). **d** FMR_oto_ relative to SMR (FMR/SMR) along the MTE prediction compared to MMR factorial scope (MMR/SMR) values given by previous studies (Test 3)

FMRs were inferred for a wild cod population from Newfoundland^30^ based on the published δ^13^C_oto_ data converted to oxygen consumption according to Eqs. 1 and 2. We define the oxygen consumption rate (mg O_2_ kg^−1^ h^−1^) estimated from δ^13^C_oto_ values as FMR_oto_. In the wild cod, FMR_oto_ decreased sharply between ages 0 and 1 (size range ca. 6–18 cm), followed by a gradual decrease with age (and size) after 1 year of age (Fig. 4a). FMR_oto_ was always higher than MTE predictions of SMR and experimental SMR values extracted from the literature (Supplementary Note 1), and the slope of FMR_oto_ against MTE-predicted SMR was close to 1 (1.19 ± 0.250) (Fig. 4b). Compared to FMR_oto_, the slope of the relationship between measured SMR from the literature and the MTE-predicted SMR was lower and departed from 1, and measured SMR values exceeded those predicted by MTE when predicted oxygen consumption rates were below approximately 75 mg O_2_ kg^−1^ h^−1^ (Fig. 4b). Moreover, FMR_oto_ was always higher than the sum of SMR and SDA, even when the maximum reported value, 0.4 times total energy intake, was used for the SDA estimation (Fig. 4c). In short, our FMR estimates fit the expectations based on the metabolic power budget and pass Test 1.

The metabolic cost of activity was evaluated using FMR_oto_ estimates and an assumed SDA of 0.12 times FMR_oto_ (an average value for juvenile cod). Using these values, activity metabolism accounted for 37−59% of FMR_oto_, depending on fish body mass and environmental temperature. This corresponds to 0.73–2.0 times SMR, which is in the range of 0.3–3.9 published by previous studies^31,32^, and hence satisfies Test 2.

The FMR_oto_ factorial scope (defined as the ratio FMR_oto_/SMR) decreased from 3.5 to 1.9 with increasing oxygen consumption and the range was consistently lower than the MMR factorial scope (MMR/SMR) observed from literature (2.3–3.5)^33,34^ (Fig. 4d). FMR_oto_ in wild fish did not approach maximum metabolic rate and individuals maintained excess metabolic capacity in the natural environment. The FMR_oto_ therefore pass Test 3. The conclusion of the three tests is that the FMR estimates are within the range of expected values. Further, these tests show that the otolith carbon isotope proxy for FMR is not limited to relative comparisons, but can be used to derive absolute estimates of FMR expressed in units of oxygen consumption rates.

### Allometric scaling exponent (*α*) estimation (Tests 4 and 5)

Ambient temperature and body mass were correlated with the proportion of metabolic carbon in otolith aragonite across 76 fish species (Fig. 2). We evaluated the allometric scaling exponent (*α*) between body size and *M*_oto_ according to Eq. 4 (a combination of Eq. 1 and oxygen consumption rates predicted from MTE). The best fit model between *M*_oto_ and body size contained a scaling exponent of −0.105, which was considerably higher than the theoretical scaling exponent for the relationship between theoretical mass-specific SMR and body size (−0.25) or that observed for fish SMR (−0.21)^6,35^, but smaller than −0.088 estimated from MMR^36^, i.e. the *M*_oto_ scaling fall between MMR and SMR body mass scaling as expected (Test 4).

**Fig. 5:**
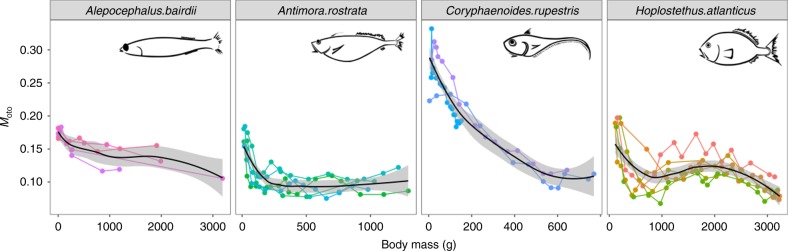
Species-specific trends of the *M* term in fish lifespan from four deep sea fish species. These four deep sea fish species are captured above 1500 m in the Northeast Atlantic. The grey area is 95% confidence interval

The final test (Test 5) is an example of how individual metabolic life histories can be reconstructed from isotope analyses of samples taken across otolith growth rings. Estimates of lifelong variation in *M*_oto_ were made for four deep sea fish species (*Alepocephalus bairdii*, *Antimora rostrata*, *Coryphaenoides rupestris* and *Hoplostethus atlanticus*, using data from Trueman et al.^37,38^ and Chung^39^). These four species displayed different ontogenetic metabolic trajectories and differences were more pronounced in the early life stages (Fig. 5). Following Eq. 4, we identified models best fitting the ontogenetic variations of the *M*_oto_ term by varying the allometric scaling exponents of body mass. The best fit exponents varied among species and *H. atlanticus* (−0.135) and *A. rostrata* (−0.169) had higher best-fit values than *A. bairdii* (−0.251) and *C. rupestris* (−0.626), implying species-specific differences in metabolic life histories.

## Discussion

The difficulty of estimating time-integrated FMR in marine fish, particularly in units of oxygen consumption rate, constrains our knowledge of how ecological and environmental factors interact to shape individual and population-level physiological performance. For example, the relationship between metabolic rate (oxygen consumption rates) and the pace of life history^28^, activity^27^, and ecosystem functioning^40^ are highly debated current topics. Our approach is a major step towards exploring and understanding these phenomena. Earlier studies have used measurements of δ^13^C_oto_ as a proxy for metabolic rate without being able to convert the values into oxygen consumption. Hence, these studies were limited to qualitative estimates of differences in FMR among life stages or groups of fish^41^ or correlative studies between δ^13^C_oto_ or *M*_oto_ and growth rate^42^. Our study calibrates the *M*_oto_ to measured oxygen consumption and provides strong evidence for the accuracy of this FMR proxy. We build on the well-established observation that there is a metabolic influence on the isotopic composition of carbon in otolith aragonite and describe the nature of the relationship between oxygen consumption rate and δ^13^C_oto_ values in laboratory-reared fish. Our calibration (Eq. 1) allows us to produce time-integrated, retrospective estimates of the oxygen consumption rate associated with FMR in individual wild fish. We applied five tests to determine whether otolith-based estimates of FMR lie within expected ranges, and our measurements passed all tests. We demonstrate the power of δ^13^C_oto_ values as a metabolic proxy, and also suggest some potential applications of δ^13^C_oto_-based investigations at individual, species, population and macroecological levels.

In Tests 1−3, we find that FMR_oto_ values fall with the range expected from theory and previous studies of SMR and MMR. The observed decrease in weight-specific FMR_oto_ with size conforms with expectations and follows the pattern of SMR. Previous studies have found that small, e.g. larval and juvenile, fish have decreased aerobic scope (MMR/SMR)^43^. Our results also show a decreasing factorial scope between FMR and SMR with increasing SMR (Fig. 4d) suggesting that smaller fish (with higher weight specific SMR) use a larger proportion of actual metabolic expenditure on SMR compared to larger fish. However, across the entire MTE-predicted range of SMR, the otolith-based estimate of FMR allowed realistic scopes for SDA and activity (Fig. 4c)^34,44^.

The relationship between metabolic components is not fixed but varies among life stages, species and environmental conditions. For example, the ratio of SDA to assimilated energy decreases with food intake^45^ but increases with temperature increases^46^, and mass-specific activity metabolism decreases with size of larval cod^32^. Moreover, the prediction of SMR based on the MTE equation accounts for 82% of the variation in oxygen consumption rates observed from our rearing experiment. In the natural environment, SMR may be altered by food availibility^47^, reducing the accuracy of SMR prediction based solely on body mass and temperature, and biasing the estimation of FMR factorial scope.

According to the MTE, the allometric scaling exponent follows 3/4-power law for whole-organism metabolic rate^5^. However, diverse metabolic exponents are found both among and within species^27^. The MLBH predicts that the scaling exponent value will vary between 2/3 and 1, depending on the dominance of thermoregulation in relation to body surface (2/3) or mass-related energy use/demand (1)^24–27^. With higher activity level, the allometric scaling exponent will trend toward 1 in relation to the increase in muscular power production. Thus, we evaluated the metabolic exponent in a wide range of teleost species by the central equation in MTE (Eq. 3) and the relationship between *M*_oto_ term and oxygen consumption. δ^13^C_oto_ field metabolic proxies give a value of 0.895 (the allometric scaling exponent of whole organisms is derived from −0.105, which is the exponent based on the mass-specific estimation). The value lies between the observed scaling exponent 0.79 from SMR^6,35^ and 0.912 from MMR^36^, corresponding to the prediction in MLBH that the active fishes have higher scaling exponents than fish in resting condition, and that scaling exponents should decrease in order from MMR to FMR and SMR. However, 39.4% of the among-species variation in the *M*_oto_ term remains unexplained, potentially indicating behavioural or physiological variation in the scaling between field and MTE-predicted SMRs, and/or different relationships between *M*_oto_ and oxygen consumption rate among species.

A comparison of metabolic variation through ontogeny and the corresponding allometric scaling exponent (*α*) was made for four deep sea fish species to explore the interspecific metabolic history and exponent values. Unsurprisingly, all species have a similar *M*_oto_ values at the adult stage because they inhabit similar depths around 1500 m and metabolism in deep sea animals is strongly related to habitat depth^48,49^. However, the metabolic trajectory in *C. rupestris* shows a noticeable and continuous decrease with size, which relates to a relatively long duration of the larval/juvenile shallow water phase in warmer environments and pelagic larvae/juveniles descending from ca. 500 to 1500 m between 8–14 years of age^39^. The estimations of the allometric scaling exponents based on Eq. 4 have already considered the temperature influence associated with ontogenetic migrations, but still give a relatively low value of 0.374 in *C. rupestris* (−0.626 estimated from mass-specific metabolic rate), which exceeds the boundary ranges given by the MLBH. Our results imply that *C. rupestris* have a relatively lower metabolic level during ontogeny compared to other species, and we further suggest that fish morphology in relation to swimming modes may explain some of the difference between the four species. Anguilliform swimming favours eel-like or elongated fish with high swimming efficiency and low metabolic demand. *C. rupestris* have a higher degree of body elongation, followed by *A. rostrata, A. bairdii*, and then the highly fusiform body of *H. atlanticus*^50^. The elongated body form facilitates efficient swimming in the deep^50^, and coincidentally, the allometric scaling exponent correlates with indices describing body elongation in these four species. Alternatively, accumulation of metabolically inert or low performing tissues during ontogeny may cause the allometric scaling exponent value to fall below 2/3^51^. The inference needs further investigation, but data imply that the otolith-based proxy for FMR can be used to relate life history traits and metabolism at the individual level, and can provide additional information of intraspecific and interspecific variation of allometric scaling exponents.

Some issues remain that need to be addressed before applying the δ^13^C_oto_ proxy to estimate FMR in wild fish. First, the scaling between the *M*_oto_ term and oxygen consumption established in this study is based on Atlantic cod, and it needs to be examined for more species to test whether it is universal or (more likely) varies between species or functional groups. For example, the highest value of *M*_oto_ term estimated from *C. rupestris* exceeds the upper bound of scaling established from Atlantic cod. Second, uncertainty in how precisely the δ^13^C_oto_ values reflect FMR should be investigated in detail. For instance, δ^13^C_DIC_ values vary in the ocean surface and coastal areas which will increase the uncertainty in *M*_oto_. Third, more precise measurements of SDA could give a better estimation of components in metabolic power budgets. Finally, acid-base regulation of respired CO_2_ transport in the plasma and excretion from the body should be evaluated for possible influences on the isotopic metabolic proxy^17,52,53^.

In our study, the temporal resolution of FMR_oto_ is scaled to yield information on the behavioural and environmental impacts on metabolic rate over a relatively short time scale (days – few weeks). We have estimated that the otolith sampling protocol used in our analyses integrates otolith growth over approximately 15 days. Temporal resolution can be decreased by drilling deeper into the otolith, thereby sampling carbonate deposited over a longer period. This will allow estimates of energy expenditure integrated over sufficiently long time periods to capture the metabolic cost of a wider range of natural behaviours (e.g. seasonal movements and reproduction). This may be desirable in order to minimise the influence of abnormal or extreme behaviours present only over a short time period. Alternatively, temporal resolution could be increased by decreasing the otolith sample amount and precisely controlling drilling depths across the growth axis by microdrilling, but ultimately the maximal resolution is dependent on the growth rate of the otolith. Hence, the method is flexible with regard to temporal resolution, although the resolution is limited by the growth rate and size of the otolith. Consequently, the method should not be seen as a panacea, but a widely applicable method complementing traditional laboratory-based approaches for exploring fish physiology under environmental change.

In summary, our methodology provides an alternative approach to investigate and compare the metabolic expression of behavioural responses to environmental changes among individuals, species, populations and geographic distributions. Current theory predicting the performance of fish under different environmental change scenarios^54,55^ typically does not take into account individual behavioural and physiological responses and adaptation, or altered metabolic costs associated with changing foraging and predation dynamics. Otolith-based measurements of individual-level FMRs in wild fish populations could provide additional data needed to enhance ecological models and provide more accurate and precise predictions of fish population dynamics, behaviour and adaptation under a long-term environmental change.

## Methods

### Temperature-controlled experiment

Atlantic cod were reared at Institute of Marine Research, Norway. Eggs were fertilised and hatched in bags at Parisvatnet field station (Norway) in March 2014. The larvae and juveniles were fed zooplankton and then dry food until reaching a size of 50–80 g in November 2014. To avoid cannibalism, the smallest and largest individuals were discarded and only fish between 50 and 80 g were included in the experiment. Fish were then transferred to the research station Matre, individually PIT-tagged (12 mm SMARTRAC smart-glass-tag), length and weight measured and assigned to one of the four triplicate temperature groups, 4 °C, 7 °C, 10 °C and 14 °C. The 10-degree temperature range covers the most of the suitable temperature range for Atlantic cod^56^. The fish were kept in 2 m^3^ tanks and were fed food pellets (Skretting’s Amber Neptun size 4 mm) from the same production cycle throughout the experiment to ensure consistent diet isotope values. The temperature in the tanks was gradually lowered or raised by 2 °C per day until the experimental temperature was reached. Water from the fjord was cooled or heated in a pipeline system and pre-mixed individually for each temperature group to ensure the exact same temperature before entering the tanks. The tanks are grouped with four groups of three tanks of equal water quality. The temperature was controlled by a heat exchanger. The temperature-controlled water entered the tanks, and the temperature was logged electronically at the entrance of each of the temperature groups. Temperature varied with a standard deviation of 0.51, 0.18, 0.16 and 0.37 for the four temperature groups (4 °C, 7 °C, 10 °C and 14 °C respectively).

Fish from each tank were sampled two or three times during the 12-month duration of the experiment. The experiment for each tank ended when the average size of the fish had reached approximately 300 g, which occurred at different dates between August and November 2015 largely determined by the rearing temperature. At termination, fish were euthanized with an overdose of benzokain and individual length (mm) and body mass (g) were recorded (Table 1).

**Table 1:** Summary of fish used in the otolith carbon isotope analyses

Temperature (°C)	Number	Weight (g)	δ^13^C_oto_ (‰)	δ^18^O_oto_ (‰)	δ^13^C_DIC_ (‰)	δ^13^C_diet_ (‰)	*M*_estimated_	Reconstructed oxygen consumption (mg O_2_ kg^−1^ h^−1^)
4	11	118–298	−2.23 ± 0.23	3.59 ± 0.21	−0.15;−0.22	−19.96 ± 0.30	0.10 ± 0.012	70 ± 6.5
7	15	111–410	−2.87 ± 0.44	2.74 ± 0.26	−0.13;−0.23	−20.13 ± 0.68	0.14 ± 0.024	93 ± 10
10	19	128–678	−3.25 ± 0.35	2.27 ± 0.29	−0.27;−0.32	−20.80 ± 0.82	0.14 ± 0.017	105 ± 16
14	18	94–530	−4.02 ± 0.33	1.50 ± 0.30	−0.21;−0.31	−21.38 ± 0.63	0.18 ± 0.015	145 ± 28
Individuals used for oxygen consumption measurements								
**Temperature (°C)**	**Number**	**Weight (g)**	**δ** ^**13**^ **C**_**oto**_ **(‰)**	**δ** ^**18**^ **O**_**oto**_ **(‰)**	**δ** ^**13**^ **C**_**DIC**_ **(‰)**	**δ** ^**13**^ **C**_**diet**_ **(‰)**	***M***_**estimated**_	**Measured oxygen consumption (mg O**_**2**_ **kg**^**−1**^ **h**^**−1**^**)**
4	4	270–406	−2.76 ± 0.37	3.15 ± 0.29	−0.31	—	—	36.7–56.6
7	5	338–710	−3.12 ± 0.63	2.97 ± 0.23	−0.27	—	—	40.0–53.6
10	4	532–783	−3.46 ± 0.40	2.34 ± 0.37	−0.23	—	—	50.1–71.0
14	4	306–631	−3.83 ± 0.33	1.44 ± 0.29	−0.11;−0.22	—	—	66.1–93.8

### Oxygen consumption measurement

At the end of the 12-month experiment 17 individuals (Table 1) were randomly chosen for intermittent flow respirometry of SMR. To estimate SMR, oxygen consumption rates were measured at different swimming speeds in a swim tunnel and then extrapolated to zero speed. This is a well-established method for estimating SMR (see review by Chabot et al.^57^). These measurements were used to establish the relationship of temperature, body mass and SMR.

The swim tunnel manufactured by Loligo systems had a volume of 87.3 l. Dissolved oxygen concentration, oxygen saturation and temperature were measured and logged using an oxygen optode 3835 from Aanderaa data instruments. In addition to the remote control and monitoring of swim tunnel instruments, live video feed of the tunnel both horizontally and vertically was obtained to monitor stress and fish behaviour.

Length measurement and transfer of the fish from rearing tank to respirometer was completed swiftly with minimum handling of the cod using nets to apply a minimum of stress to the animal. After transfer to the swim chamber, flow was kept at a steady pace of 0.2−0.4 BL s^−1^ and manually adjusted for a while when the fish was learning how to swim correctly in the swim tunnel. When the fish had figured out the direction and held a steady pace for a while, the flow rate was set to a standard of 0.2 BL s^−1^ for measurements after a 24 h acclimatisation during which the fish were not fed.

During the experiment, we used a loop protocol of 50 s wait period and 4-min measuring period followed by a 10-min flush period. Oxygen uptake recorded during periods of unwanted behaviour was left out due to potentially elevated oxygen consumption rates not representing actual base line MO_2_ rates at the certain swimming speed. Unwanted behaviour was characterised as the cod resting with the tail placed on the hind grid or the fish laying sideways on the hind grid in the swimming chamber. A few of the individual cod measured during this experiment would have the tendency to turn within the swimming chamber regularly but it was decided that this behaviour would only be characterised as unwanted if the cod would touch the hind grid, which in most cases was not so.

In the measuring period a short initial lag period was observed with increasing O_2_ concentrations in the first 50 s. Hence, measurements in the first 50 s were excluded from all loops measuring oxygen consumption. This initial lag period represents the wait period necessary for a linear relationship between oxygen uptake and time to be established. The continuous output in oxygen percentage with additional temperature and salinity data was converted to a unit in micromoles per litre seawater using the equation of Garcia and Gordon^58^.

Only the last two of the three loops at each swimming speed was used to calculate MO_2_. In the case of inadequate measurements in second or third loop due to unwanted behaviour, the MO_2_ in the remaining loop was used. Oxygen consumption was measured at 0.2−1.2 body lengths per second (BL s^−1^) in 0.2 BL s^−1^ intervals. An exponential model was fitted to the data and SMR was estimated by extrapolating oxygen consumption to 0 BL s^−1^ using an ordinary least squares regression.

### Isotope analyses

Seawater in the tanks was from the costal water off Bergen and kept as continuous flow during the experiment. Water samples from each tank were periodically collected and analysed for δ^13^C of DIC by using a Thermo Scientific Delta V plus Isotope Ratio Mass Spectrometer (IRMS) connected to a Gasbench at Farlab, Department of Geoscience, University of Bergen. Five drops of 100% phosphoric acid was added to Exetainers (Labco, Lampeter, UK), capped and flushed with He (5.0 quality). One millilitre of water sample was added to each exetainer, allowed to equilibrate (>18 h, 24.0 °C) before measurement of CO_2_ from the headspace. Carbon isotope ratios of water DIC (δ^13^C_water_) are reported in delta notation relative to VPDB. Precision and accuracy monitored through repeat analyses of laboratory standards and samples were less than 0.1 per mil (‰).

Otoliths collected from fish in the reared experiment were cleaned with 0.2 M NaOH for 30 min followed by 1 min in a ultrasonic bath, washed by MilliQ water three times, dried in an oven 30 °C for 48 h and then stored in vials. Cleaned otoliths were then drilled to obtain powder for determining isotope ratios (δ^13^C and δ^18^O values) of the aragonite. The drilling area was controlled and made up 3.5 mm^2^ on the distal surface of otoliths and the average weight of otolith powder sample was 120 μg reflecting 10–15 days of otolith growth. To improve accuracy of isotopic values, 2−3 samples were obtained from different drilling areas on each otolith and the average isotopic value was used further on. Samples were analysed using a Thermo Scientific Delta V plus IRMS equipped with GasBench II at the Center for Geomicrobiology, Aarhus University, and delta notation was relative to VPDB (Vienna Pee Dee Belemnite). Precision and accuracy monitored through repeat analyses of carbonate standards were better than 0.1 per mil (‰).

Our method allows sampling of well-defined, small areas of the otolith without destroying the whole otolith. This means that we can sample aragonite for analysis from birth to death. In principle, a continuous trajectory of samples can be obtained with a resolution depending on the growth rate and size of the otolith. Thus, repeated sampling in one otolith at different life stages can be conducted by micromiling as shown for the four deepwater fishes.

After drilling, each otolith went through the previous cleaning procedure again and dried in an oven. The dietary carbon isotope signal of fish was determined by the otolith organic matters and extraction of otolith organic matters was following the well-established protocol from Grønkjær et al.^59^. Samples were analysed using Thermo Scientific Delta V plus IRMS at the Center for Geomicrobiology, Aarhus University, and delta notation was relative to VPDB (Vienna Pee Dee Belemnite). Because the whole otolith was used in this analysis, the δ^13^C_diet_ value may be biased from materials deposited before the temperature controlling experiment. Thus, otoliths collected before temperature assignment were analysed and compared to samples during temperature-controlled experiment.

### Temperature and *M*_oto_ estimated from δ^18^O and δ^13^C values

Otoliths from the rearing experiment were collected for carbon and oxygen isotope analyses (*n* = 80). The individual information is summarised in Table 1. Based on the regression of the four temperatures on otolith δ^18^O values, we evaluated the individual temperature variation from a Monte Carlo simulation. We used the temperature inferred by otolith δ^18^O values for each reared cod individual in the following analyses.

The proportion of metabolically derived carbon in otolith carbonate (*M*_oto_) can be estimated as:

$\updelta ^{13}{\mathrm {C}}_{\mathrm {{oto}}} = M_{{\mathrm {oto}}} \times \updelta ^{13}{\mathrm {C}}_{{\mathrm {diet}}} + \left( {1 - M_{{\mathrm {oto}}}} \right) \times \updelta ^{13}{\mathrm {C}}_{{\mathrm {DIC}}} + \varepsilon$, or expressed as:

2 $$M_{{\mathrm {oto}}} = \frac{{\left( {\updelta ^{13}{\mathrm {C}}_{{\mathrm {oto}}} - \updelta ^{13}{\mathrm {C}}_{{\mathrm {DIC}}}} \right)}}{{\left( {\updelta ^{13}{\mathrm {C}}_{{\mathrm {diet}}} - \updelta ^{13}{\mathrm {C}}_{{\mathrm {DIC}}}} \right)}} + \varepsilon,$$

where δ^13^C_diet_ and δ^13^C_DIC_ are the average δ^13^C values of the diet and DIC in seawater and the *ε* term is the total net isotopic fractionation during carbon exchange between DIC and blood and between blood and endolymph in which the otolith is formed^17,21,38^. The ε term was set as 0 in this study following the finding in Solomon et al.^17^.

### Routine metabolic rate determination of reared cod

SMR scales predictably with temperature and body mass across fish species^5,60^:

3 $${\mathrm {Metabolic}}\,{\mathrm {rate}} = B_0 \times \left( {{\mathrm {Body}}\,{\mathrm {mass}}} \right)^\alpha \times e^{ - \frac{{0.65}}{{\left( {8.62 \times 10^{ - 5}} \right) \times \left( {{\mathrm {Kelvin}}\,{\mathrm {temperature}}} \right)}}},$$

where the *B*_0_ is the normalised constant and *α* is the allometric scaling exponent of body mass, which follows 3/4-power law in MTE (as −0.25 for mass-specific metabolism)^5^ but is found to be 0.79 for teleost fishes^6,35^. From the 17 reared individuals with measured oxygen consumption, we established the relationship between oxygen consumption, and body mass and temperature from Eq. 3. Based on this relationship, SMR of other 63 juvenile cod collected during 1-year reared experiment were estimated with known body size and δ^18^O-deduced experienced temperature. Then, RMR_fed_ was calculated as 1.55 (±0.0832)×SMR, which was obtained from experimental estimations of fish living at 6 (1.49~1.65) and 14 °C (1.44~1.57) by Jordan et al.^29^.

### Five tests of δ^13^C_oto_ as a practicable metabolic proxy

We evaluated the power and suitability of our FMR_oto_ protocol using three different datasets. The first three tests were conducted using published otolith data from a wild cod population from Newfoundland^30^. We estimated FMR from the δ^13^C_oto_ proxy to test whether FMR values fall in a reasonable range given by the metabolic power budget. According to the metabolic power budget, the FMR of fish is the sum of the SMR, activity and SDA^61^. FMR in wild cod should therefore be higher than the sum of SMR and SDA. We tested this by assuming that FMR reflects total time averaged energy intake, that SDA is 0.1–0.4 times total assimilated energy^46,62–64^, and growth is negligible over the short time period captured in a single otolith isotope measurement in adult fish (Test 1). Then, we compared our FMR estimates to published estimates of cod activity metabolism and aerobic scopes. FMRs of wild cod reported in the literature lie between 0.3 and 3.9 times SMR^31,32^ (Test 2); and because fish seldom reach the maximum metabolic rate in the field, the FMR factorial scope (FMR/SMR) in wild juvenile cod should be lower than the maximum metabolic rate factorial scope (MMR/SMR), which has values of 2.3–3.5^34,46^ (Test 3).

Metabolic rates show an allometric scaling with body mass, expressed in the allometric scaling exponent, and diverse metabolic scaling exponents have been observed across fish taxa, species and life stages^24,26^. The fourth and fifth tests evaluated allometric scaling exponents estimated from the δ^13^C_oto_ proxy following the assumptions of the MLBH^24–27^. According to the MLBH, the scaling exponent value is expected to vary between 2/3 and 1, depending on the dominance of thermoregulation in relation to body surface (2/3) or mass-related energy use/demand (1)^24–27^. MLBH proposes that very active fishes (i.e. with metabolic rates close to MMR) have higher scaling exponents than fish in resting condition (SMR)^65^, because the muscle energy expenditure associated with swimming is related to body mass resource demand, which will increase the scaling exponent toward 1^26^. Therefore, the metabolic scaling exponent estimated from FMR should fall between derived values for the scaling of MMR and SMR with body mass. To test the assumption, we used literature-based data from multiple fish species to estimate the metabolic scaling exponent at across species. Then, we compared the values of exponents estimated from SMR, FMR (derived from the δ^13^C_oto_ proxy) and MMR (Test 4). Finally, the metabolic-level boundary hypothesis predicts that species of fish with different life history and functional traits will exhibit different allometric scaling between FMR and body mass^24–27^. For example, pelagic and benthopelagic fish have a lower value of metabolic scaling exponent compared to benthic and bathyal species^27^. In the fifth test, we used sequential sampling of δ^13^C_oto_ values in otoliths of four deep sea fish species^38,39^ to evaluate the allometric scaling exponent among species (Test 5).

*Test 1*: *FMR*_*oto*_ *is higher than the sum of SMR and SDA*. Initially, we reconstructed individual FMR of a wild cod population from Newfoundland^30^. To determine the *M*_oto_ term, δ^13^C_oto_ and δ^13^C_diet_ (muscle ^13^C signals) values were extracted from Jamieson^30^ and δ^13^C_DIC_ value was assumed to be 1.28 ± 0.36‰^66^. FMR_oto_ was estimated by the relationship between the *M*_oto_ term and oxygen consumption from our reared Atlantic cod. According to the metabolic power budget, the total metabolic rate of fish (i.e. FMR) is composed by SMR, activity and SDA^61^. SDA is dependent on the amount of energy intake and account for 10–40% of assimilated energy^46,62–64^, and we assumed FMR_oto_ reflects the total energy assimilated. We determined SDA as 40% of FMR_oto_, considering the maximum contribution of SDA in the metabolic power budget. SMR of the fish was determined by body mass and environmental temperature, following the relationship between body mass, temperature and SMR across previous studies on Atlantic cod (Supplementary Note 1). Experienced temperature of cod was reconstructed by otolith δ^18^O values (extracted from Jamieson^30^) based on the relationship established by Campana^67^.

*Test 2: Activity metabolism is 0.3–3.9 times SMR*. Activity metabolism was calculated as the difference between FMR_oto_ and the sum of SMR and SDA. SMR determination was following the descriptions above, and SDA was 12% of FMR_oto_, which was the averaged values found in the previous study on juvenile cod (an average of 8−16%^46^).

*Test 3: The FMR factorial scope is lower than MMR metabolic scope*. FMR_oto_ and SMR acquired from the previous estimation were used to determine FMR factorial scope (FMR/SMR), and the values of MMR metabolic scope in juvenile cod was obtained from Soofiani and Priede^33^ and Schurmann and Steffensen^34^. Due to the different range of fish body mass and temperature across data from this study and literature, all the comparisons in these three tests were made against MTE-based SMR predictions (Fig. 2).

*Test 4: The allometric scaling exponent derived from FMR falls in between the values of exponents from SMR and MMR*. δ^13^C_oto_ values of 76 fish species were extracted from 24 studies. We calculated *M*_oto_ values using a mean δ^13^C_DIC_ value of 1 ± 0.5‰ and a δ^13^C_diet_ value of −19 ± 3‰, respectively. The *M*_oto_ values were compared to reported fish body size and experienced temperature. If the temperature was not provided in the literature, we used otolith δ^18^O values to reconstruct temperature. Combining Eqs. 1 and 3, we get:

4 $$M_{{\mathrm {oto}}} = C\left( {1 - e^{ - k\left( {B_0 \times \left( {{\mathrm {Body}}\,{\mathrm {mass}}} \right)^\alpha \times e^{ - \frac{{0.65}}{{\left( {8.62 \times 10^{ - 5}} \right) \times \left( {{\mathrm {Kelvin}}\,{\mathrm {temperature}}} \right)}}}} \right)}} \right).$$

The metabolic scaling was examined based on Eq. 4. We varied *α*, and modelled *M*_oto_, body mass and temperature from 76 fish species to find the *α* that yielded the best fit model i.e. the lowest AIC. The *α* value from the best fit model was reviewed as the allometric metabolic exponent derived from the FMR.

*Test 5: The allometric scaling exponent derived from FMR varies between species*. Data for four deep sea fish species (*Alepocephalus bairdii, Antimora rostrata, Coryphaenoides rupestris and Hoplostethus atlanticus*) were extracted from Trueman et al.^37,38^ and Chung^39^. The *M*_oto_ term was determined using a δ^13^C_DIC_ value of 1 and δ^13^C_diet_ values from muscle isotopic analyses. The body mass of fish during ontogeny was back-calculated from otolith increment widths and the thermal history was reconstructed by otolith δ^18^O values. This information was provided by Trueman et al.^37,38^ and Chung^39^. Following Eq. 4, we modelled body mass and the *M*_oto_ term variation with changing *α* values to find the best fit model with lowest AIC.

In this study, the MTE predictions, *M*_oto_ term determinations and FMR_oto_ estimations were conducted by Monte Carlo simulation with 10,000 repeats, which took uncertainty from every variables into account. All the statistics, such as linear regression models, non-linear regression models, ANOVA and Monte Carlo simulations, were performed by R^68^, and figures were produced using the package ggplot2^69^.

## Supplementary information

Supplementary Information

## Data Availability

The datasets generated and/or analysed during the current study are available in the Dryad repository^70^, 10.5061/dryad.1hg55vm
