# Supplementary material for: Field metabolic rates of teleost fishes are recorded in otolith carbonate
Source: Commun Biol. 2019 Jan 18;2:24. doi: 10.1038/s42003-018-0266-5 (PMC6338665; doi:10.1038/s42003-018-0266-5)
Supplement: Supplementary file 1 — Supplementary Information [file 42003_2018_266_MOESM1_ESM.pdf]

Supplementary Note

- *Metabolic theory of ecology (MTE) prediction*

$$Metabolic\ rate\ = B_0 \times (Body\ mass)^{\alpha} \times e^{-\frac{0.65}{(8.62\times10^{-5})\times(Kelvin\ temperature)}}$$

where the B_0 is the normalised constant and α is the allometric scaling exponent of body mass. Following the core equation of MTE, oxygen consumption of Atlantic cod with specific body mass and temperature can be estimated with $2.03*10^{13}$ of B_0 (which is acquired from our reared experiment) and 0.79 of α (Killen et al. 2010). For 76 species extracted from literature, MTE prediction is based on $1.64*10^{13}$ of B_0, which is recalculated from Killen et al. (2010), and 0.79 of α (Clarke & Johnston 1999; Clarke 2006).

- *Estimations of sample number demand in a comparison of FMR_{oto} between two wild cod population according to the method developed in this study.*

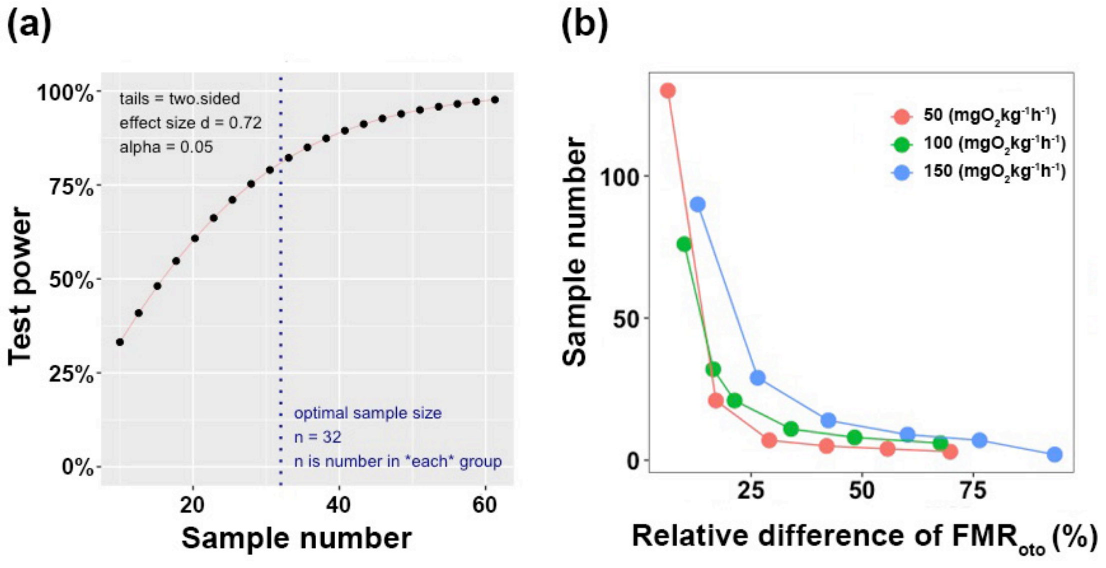

The estimation is conducted with R package “pwr” (https://github.com/heliosdrm/pwr). (a) The minimum sample numbers vary with test power, which is calculated as 1- β (β: the probability of type II error). The case is 17% of FMR_{oto} difference between two wild cod populations and FMR_{oto} of population in lower metabolism is 100 ($mgO_2kg^{-1}h^{-1}$). The effect size is calculated from uncertainty (standard deviation) in our method. (b) The minimum sample number is changing according to specific FMR_{oto} values and relative difference between two populations. Different colours indicate a FMR_{oto} value of the population with lower metabolic rate.

- *Determination of the allometric scaling exponent of body mass (α)*

Combining the increasing exponential decay model and MTE prediction equation:

$$M = C\left(1 - e^{-k\left(B_0 \times (Body\ mass)^{\alpha} \times e^{-\frac{0.65}{(8.62\times10^{-5})\times(Kelvin\ temperature)}}\right)}\right)$$

If the α is set as 0, the model between M variation and MTE prediction will give a value of AIC. Then we change α from -1 to 0, it will give us a series of AIC values. The α corresponding to the lowest AIC values is supposed to be the best model fit value. R codes are shown below:

```
o<-700 # data number
p<-1001
alpha<-seq(-1, 0, by=0.001)
MTE<-matrix(NA, ncol=p, nrow=o)
AICalpha<-NA
Mass<- #Fish body mass
Temp<-#Ambient temperature
M<-#the estimated M term
```

```
for(i in 1:o){
  for(j in 1:p){
  MTE[i,j]<-1.641894e+13*((Mass[i]/1000)^(alpha[j]))*(exp(-0.65/((8.62*10^-
5 )*(273.15+Temp[i]))))
}
}

for(j in 1:p){
    AICalpha[j]<- AIC(nls(M~ C*(1-exp(-k*MTE[,j])),
                        start=list(C=0.6, k=0.069083)))
  }
```

- *Standard metabolic rate (SMR) of Atlantic cod extracted from literature:*

$$SMR = (2.02 \times 10^{13}) \times (Body\ mass)^{-0.21} \times e^{-\frac{0.65}{(8.62\times10^{-5})\times(Kelvin\ temperature)}}$$

Bodymass.g	Bodylength.cm	Temperature	SMR.measured (mg O_2 $kg^{-1}h^{-1}$)	Reference
1.53	NA	10	115.2	(Herbing & White 2002)
2.53	NA	10	76.8	(Herbing & White 2002)
3.45	NA	10	76.8	(Herbing & White 2002)
5.17	NA	10	76.8	(Herbing & White 2002)
7.81	NA	10	76.8	(Herbing & White 2002)
39	NA	15	145	(Soofiani & Hawkins 1982)
56	NA	18	144	(Soofiani & Hawkins 1982)
59	NA	7	84	(Soofiani & Hawkins 1982)
61	NA	10	131	(Soofiani & Hawkins 1982)
124	NA	5	35	(Schurmann & Steffensen 1997)
140	NA	15	130	(Saunders 1963)
150	NA	10	103	(Soofiani & Priede 1985)
150	NA	15	132	(Soofiani & Priede 1985)
152	26	4	66	(Bushnell et al. 1994)
155	NA	4	61	(Steffensen et al. 1994)
158	NA	10	57	(Schurmann & Steffensen 1997)
166	NA	15	78	(Schurmann & Steffensen 1997)
242	31	5	48	(Schurmann & Steffensen 1997)
298	NA	15	74	(Schurmann & Steffensen 1997)
371	32	10	62	(Schurmann & Steffensen 1997)

1140	NA	5	39	(Saunders 1963)
1150	NA	10	58	(Saunders 1963)

References

Bushnell, P.G. et al., 1994. Exercise metabolism in two species of cod in arctic waters. *Polar Biology*, 14(1), pp.43–48.

Clarke, A., 2006. Temperature and the metabolic theory of ecology. *Functional Ecology*, 20(2), pp.405–412.

Clarke, A. & Johnston, N.M., 1999. Scaling of metabolic rate with body mass and temperature in teleost fish. *Journal of Animal Ecology*, 68(5), pp.893–905.

Herbing, I.H. & White, L., 2002. The effects of body mass and feeding on metabolic rate in small juvenile Atlantic cod. *Journal of fish biology*, 61(4), pp.945–958.

Killen, S.S., Atkinson, D. & Glazier, D.S., 2010. The intraspecific scaling of metabolic rate with body mass in fishes depends on lifestyle and temperature. *Ecology letters*, 13(2), pp.184–193.

Saunders, R.L., 1963. Respiration of the Atlantic Cod. *Journal of the Fisheries Research Board of Canada*, 20(2), pp.373–386.

Schurmann, H. & Steffensen, J.F., 1997. Effects of temperature, hypoxia and activity on the metabolism of juvenile Atlantic cod. *Journal of fish biology*, 50(6), pp.1166–1180.

Soofiani, N.M. & Hawkins, A.D., 1982. Energetic costs at different levels of feeding in juvenile cod, Gadus morhua L. *Journal of fish biology*, 21(5), pp.577–592.

Soofiani, N.M. & Priede, I.G., 1985. Aerobic metabolic scope and swimming performance in juvenile cod, Gadus morhua L. *Journal of fish biology*, 26(2), pp.127–138.

Steffensen, J.F., Bushnell, P.G. & Schurmann, H., 1994. Oxygen consumption in four species of teleosts from Greenland: no evidence of metabolic cold adaptation. *Polar Biology*, 14(1), pp.49–54.
